# A novel approach for APT attack detection based on feature intelligent extraction and representation learning

**DOI:** 10.1371/journal.pone.0305618

**Published:** 2024-06-24

**Authors:** Cho Do Xuan, Nguyen Hoa Cuong

**Affiliations:** Faculty of Information security, Posts and Telecommunications Institute of Technology, Hanoi, Vietnam; Leeds Beckett University, UNITED KINGDOM

## Abstract

Advanced Persistent Threat (APT) attacks are causing a lot of damage to critical organizations and institutions. Therefore, early detection and warning of APT attack campaigns are very necessary today. In this paper, we propose a new approach for APT attack detection based on the combination of Feature Intelligent Extraction (FIE) and Representation Learning (RL) techniques. In particular, the proposed FIE technique is a combination of the Bidirectional Long Short-Term Memory (BiLSTM) deep learning network and the Attention network. The FIE combined model has the function of aggregating and extracting unusual behaviors of APT IPs in network traffic. The RL method proposed in this study aims to optimize classifying APT IPs and normal IPs based on two main techniques: rebalancing data and contrastive learning. Specifically, the rebalancing data method supports the training process by rebalancing the experimental dataset. And the contrastive learning method learns APT IP’s important features based on finding and pulling similar features together as well as pushing contrasting data points away. The combination of FIE and RL (abbreviated as the FIERL model) is a novel proposal and innovation and has not been proposed and published by any research. The experimental results in the paper have proved that the proposed method in the paper is correct and reasonable when it has shown superior efficiency compared to some other studies and approaches over 5% on all measurements.

## 1. Introduction

### 1.1. Attack APT: Challenges and solutions

An APT attack is defined by three factors: Advanced; Persistent; Threat. This is one of the most dangerous cyber-attack techniques today. This attack technique is also known as a targeted cyber-attack. The targets of APT attacks are often government organizations, vital national agencies, or large economic and commercial groups [1,2]. The difference and dangers in the APT attack process are described in the studies [1–3] in detail and clearly. It can be seen that this difference makes this attack technique able to bypass most of the surveillance of traditional intrusion detection and prevention systems [3]. Currently, the studies on APT attack detection are relatively developed and have many different approaches [4–7]. However, the most popular and effective approach is still to combine techniques analyzing abnormal behaviors on network traffic datasets, and machine learning or deep learning algorithms [8–11]. According to the Network Traffic-based APT attack detection approach, previous studies often focused on two main solutions: i) Analyzing Network Traffic into different components such as DNS log [12,13], HTTP log [14], TLS log, etc., and then trying to detect abnormal behaviors of APT attack on each of these components [5,6], or building the behavior profile of each APT IP based on the correlation between the above components [15–22]; ii) Analyzing Network Traffic into flow or NetFlow and then extracting abnormal behaviors of APT attack. Especially, in the past time, studies [8–10,23] proposed approaches to detect APT based on building behavior profiles. Instead of directly using specific behaviors of APT, these approaches focused on extracting features of data to build behavior profiles of an attack campaign. After obtaining behavior profiles, researchers sought to synthesize and extract attributes using machine learning or deep learning algorithms. However, in studies [4,24–26], two major disadvantages of these approaches were listed as follows:

i) **The abnormal behaviors of APT attacks haven’t been built and synthesized yet**. The reason is that these approaches used available cyber-attack datasets such as DARPA/KDD Cup99, CAIDA, NSL-KDD, ISCX 2012, and UNSW-NB15 as experimental datasets. However, these datasets are often only suitable for traditional network attack techniques, so using them for APT attack detection is not reasonable. In addition, they only use pure machine learning and deep learning techniques to extract anomalous behaviors, so they are unable to extract meaningful and important information in the APT’s lifecycle.

ii) **It is difficult to detect the APT attack in imbalanced datasets**. Research [4] shows that one of the biggest difficulties and challenges in finding and detecting the signs and behaviors of APT attacks is the lack of data. To detect APT attacks, most monitoring systems have to search, extract and synthesize APT behaviors from many different sources. In these sources, the number of normal data is often many times larger than the number of anomalous data. To resolve this situation, previous approaches generated numerically balanced datasets or performed classifications without using data-balancing techniques. As a result, the classification efficiency was not high.

### 1.2. Proposed solutions

#### a) Proposed content in the paper

To resolve the above problems, in this study we propose a novel approach that combines FIE and RL. In which the FIE technique uses a combination of two main models, BiLSTM and Attention. And RL is a combination of 2 methods: Dropout for rebalancing data and contrastive learning for classification. Accordingly, two main tasks in the proposed FIERL model are:

#### Optimized extraction of abnormal behaviors of APT IPs based on FIE

This process is conducted in 2 main phases:

- Phase 1: Aggregating and extracting information of IP in network traffic using the BiLSTM network.
- Phase 2: Evaluating and highlighting important information about IP features based on the Attention network.

#### Improving the efficiency of APT IP classification using RL

The two main stages proposed in this task include:

- Phase 3: Data generation by using the Dropout method: Based on the vector obtained in phase 2, we proceed to generate additional data containing the features of APT attack to balance the number of labels in the experimental dataset by the Dropout method.
- Phase 4: APT IP classification by using contrastive learning: The Contrastive learning method has the function to find the feature vectors of APT attacks that have similarities and contrasts in the dataset. Then, pairs of similar data can be "pulled" together to learn higher-level features of each other, and conversely, contrasting pairs of data can be "pushed" away. With this approach, we will optimize the APT IP classification process.

#### b) The scientific basis of the proposed method

Based on the analysis in section 1.1 and the contents described in the operation process of the FIERL model, we believe that this study will solve the 2 major disadvantages listed above as follows:

**i) About the problem "The abnormal behaviors of APT attacks haven’t been built and synthesized yet":** In this paper, instead of using only individual deep learning models or traditional deep learning networks, we propose an APT IP aggregation and extraction method using the FIE model. With the flexible combination of BiLSTM and Attention networks in the FIERL model, we inherit and promote the advantages of each network for synthesizing and extracting important and meaningful information of APT IP based on network flow in network traffic. Specifically, we take advantage of the strength of BiLSTM, which is the ability to learn and memorize in two dimensions, to extract long‐distance features. Then, the Attention network with other components helps to highlight important and meaningful information, instead of just extracting by averaging like traditional approaches. With the support of these two advantages, the proposed FIERL model will surely build successfully and fully the behavioral profile of each APT IP in Network Traffic, thereby helping surveillance systems to identify new campaigns of APT attacks.

**ii) About the problem "APT attack detection in imbalanced datasets"**: Obviously, with the use of advanced techniques such as data generation and contrastive learning, the FIERL model will certainly be better than other models in the ability to classify APT IP. Specifically, with the support of the Dropout network, the APT attack feature vectors will be additionally generated to balance the experimental dataset. Thus, our approach is different from traditional methods. Instead of just using balanced datasets on the number of normal IPs and APT IPs, we build unbalanced datasets and then use new techniques to reconstruct the structure of the dataset to generate a new dataset. Our approach is perfectly suited to the actual task of surveillance systems because the number of APT IPs is many times smaller than the number of normal IPs in the IP set collected by systems in practice. Next, the proposal to use contrastive learning will be a big step forward for abnormal behavior classification problems. The training process of this technique has many differences and optimizations compared to traditional deep learning and machine learning techniques. Contrastive learning is being considered as the trend of recognition and classification problems. Therefore, the combination of Dropout and contrastive learning techniques will help the surveillance system not only accurately recognize APT IPs, but also reduce the rate of false predictions of normal IPs.

### 1.3. Contribution of paper

Proposing the FIERL model - a novel approach as well as a novel combination model that has not been proposed for the task of detecting APT attacks based on network traffic by any research. With this proposal, the approach in the paper has not only improved the efficiency of the APT attack detection process but also opened up a new approach to the problem of feature extraction and data imbalance.

Proposing a method to optimize the APT IP feature selection and extraction process based on the FIE model. With the flexible combination of BiLSTM and Attention models, we have succeeded in building, synthesizing, and highlighting the important information about APT IPs, thereby helping to improve the efficiency of the classification process. The FIE model is a new combination direction and no research has proposed and applied it.

Proposing the RL method for the APT IP classification task. This is an RL model with a combination of Dropout and contrastive learning that has not been proposed and applied by any research. With this proposal, the study has contributed to improving somewhat the disadvantages of the existing approaches as well as solving the problem of the datasets for APT attack detection. The experimental results in the paper have proved the role and scientific significance of the proposed RL method in the paper.

## 2. Related works

Pengfei et al. [27] suggested applying the combined CNN-LSTM model for extracting features in order to detect network anomalies from CICIDS 2017 dataset. The authors also compared their method with some other methods. As a result, the CNN-LSTM model yielded the best results on all measures. However, we think that APT attack detection based on the CICIDS2017 dataset was not reasonable. At the same time, the authors only concluded whether there was an APT attack or not based on the components of the CICIDS2017 dataset, but didn’t conclude which IP belonged to the APT attack and which IP was normal. Similarly, the study [25] proposed the CNN-LSTM combined model for detecting APT attacks based on network traffic by analyzing and evaluating the flow’s anomalous behaviors. We evaluate that this approach only provided a mechanism to extract flow behaviors, but it had no basis to conclude APT IP. Therefore, this approach was very good but needed to improve the way to conclude APT IP. Cho et al. [4] introduced the BiLSTM-GCN combined deep learning model, which combinates BiLSTM and Graph Convolutional Networks (GCN), for detecting APT attacks. The authors compared the experimental results of this model with that of Multilayer perceptron (MLP), GCN, and found that the BiLSTM-GCN model brought the highest efficiency on all metrics. This was a relatively good approach when using deep learning graph networks for the task of representing information of APT IP. However, this study didn’t propose the APT feature synthesis method based on the flow network. Besides, the way to extract features of the flow network was relatively simple, so it lost a lot of important information on APT IP via the flow network. Therefore, in our study, we will improve the approach of this paper.

The HERCULE model, which was introduced by Kexin et al. [28], analyzed and evaluated logs to detect APT attacks. This model compared the collected logs with the available logs to build multidimensional weighted graphs. As a result, it monitored and successfully detected 15 known attack campaigns. With this same idea, the MIC [29] model also analyzed monitoring logs to detect anomalies and APT attacks, but used the cause-effect inference method. This model yielded good results: recover causality with FP and FN of 0% for most programs, and only 8.3% FP and 5.2% FN in the worst results. In addition, the study [30] developed a multi-layer analysis technique using Hidden Markov Models for APT attack detection. In this, the Hidden Markov model was used to analyze and appraise correlations of alerts, thereby determining whether the sample was an APT attack or not. This model brought good results with the lowest accuracy of 91.80%, and the accuracy of predicting the next step of the APT campaign was 66.50%, 92.70%, and 100% respectively with two, three, and four correlated warnings. Lajevardi et al. [20] proposed using low-level interception and correlating operating system events with network events based on the semantic relationships defined between the entities in system ontology. Ghafir et al. [31] built a MAPT model consisting of 3 main phases for APT attack detection: detecting threats, calculating the correlation of alerts, and predicting attacks. The authors tested using some machine learning algorithms in this model such as Decision Tree, K-nearest Neighbor (KNN), Support Vector Machines (SVM), Ensemble, and brought the best accuracy result of 84.8%. Cho [6] proposed using a behavior profile analysis technique to detect APT attacks based on network traffic. Specifically, this method analyzed network traffic into different components such as DNS log, TLS, HTTP and then calculated correlations of these components, and used machine learning algorithms to evaluate behavior profiles. After testing with Random Forest (RF), Support Vector Machines (SVM), and MLP algorithms, the paper concluded that the RF algorithm was more efficient than others. However, we believe that using the flow network to conclude APT IP was still simple, leading to quite large errors. According to the approach in the paper, the authors only concluded that the flows were malicious or normal, but didn’t have a method to conclude APT IP. Cuong et al. [24] introduced the MIG model combining 3 components: MLP, Inference, and GCN. Which, the MLP network had the function of aggregating and extracting APT IP’s features, and the Inference network had the function of calculating and representing relationships of flows. Finally, GCN had the function of aggregating, extracting, and classifying APT IP and normal IP. The experimental results showed that the author’s approach partly solved the problems of studies [4,6,25]. However, we found that although using the Inference network to calculate and represent the relationship between flows brought good results, it still needs improvement because the coefficients of this network were still fixed and there was no basis for selection. In this paper, we will propose a new model that will solve the problems of this network. In the study [32], Cho proposed several Attention networks for the task of synthesizing and highlighting APT IP’s features for the first time. Accordingly, the authors proposed some combined deep learning networks and Attention networks to build behavior profiles of APT attacks in the system. Experimental results showed that with the support of Attention networks, the prediction models increased the correct prediction rate of APT IP and reduced many false predictions. With this proposal, the authors improved some shortcomings of the proposal [25]. With the same approach of using the Attention network to build behavior profiles of APT attacks, in the study [33], the authors proposed a model combining deep learning networks with Attention networks to classify APT malware. However, we found that this model was still relatively complicated and cumbersome, and was difficult to implement in practice. In addition, studies [34,35] also presented some approaches to detect APT malware using deep learning graph networks. The experimental results showed that deep learning networks were relatively effective in representing the relationship between components of APT attack behavior. However, when applying these models to real-life systems, it had performance and data problems. Therefore, these studies only work well in theory and need to have techniques to optimize computational efficiency.

In the study [36], Zhu et al. proposed a method to detect APT malware on workstations using Mitre attack. Accordingly, the authors used a technique to collect anomalous behavior of APT malware on the operating system kernel of Linux Hosts and then used the Mitre attack to detect abnormal behavior based on processes. This is one of the approaches for intrusion detection and prevention systems. Similarly, Na-Eun Park [37] also presented an approach for detecting APT malware on Mitre attack and Google Rapid Response. In addition, the study [38] proposed the Impulsive Artificial Defense method for APT malware on the workstation. Another approach for detecting APT malware on workstations using the Windows operating system has been proposed by Rory Coulter [39] in his research. Accordingly, the method proposed in the paper outperformed all techniques in reducing the false positive rate and the true positive rate by 80%. In the study [40], the authors proposed an APT attack detection method using data analysis and Metric Learning techniques. Accordingly, the authors took advantage of the method of using the origin graph to collect the execution traces of the servers to detect anomalous behavior. Besides, in the experimental part, the authors compared their proposed method with other methods, and found that the author’s method outperformed the average by 11.3% and increased the average true positive rate to 18.3%. Research [41] proposed an APT attack detection method with two stages. Firstly, a list of suspicious measures was detected. Secondly, the flow networks were extracted according to 5 features and then further analyzed to detect anomalies. In the experimental part, the authors compared their method with some other approaches and methods. Experimental results showed that the model proposed in the paper was more effective.

## 3. Our approach to APT attack detection

### 3.1. The flow of the approach

**Fig 1:**
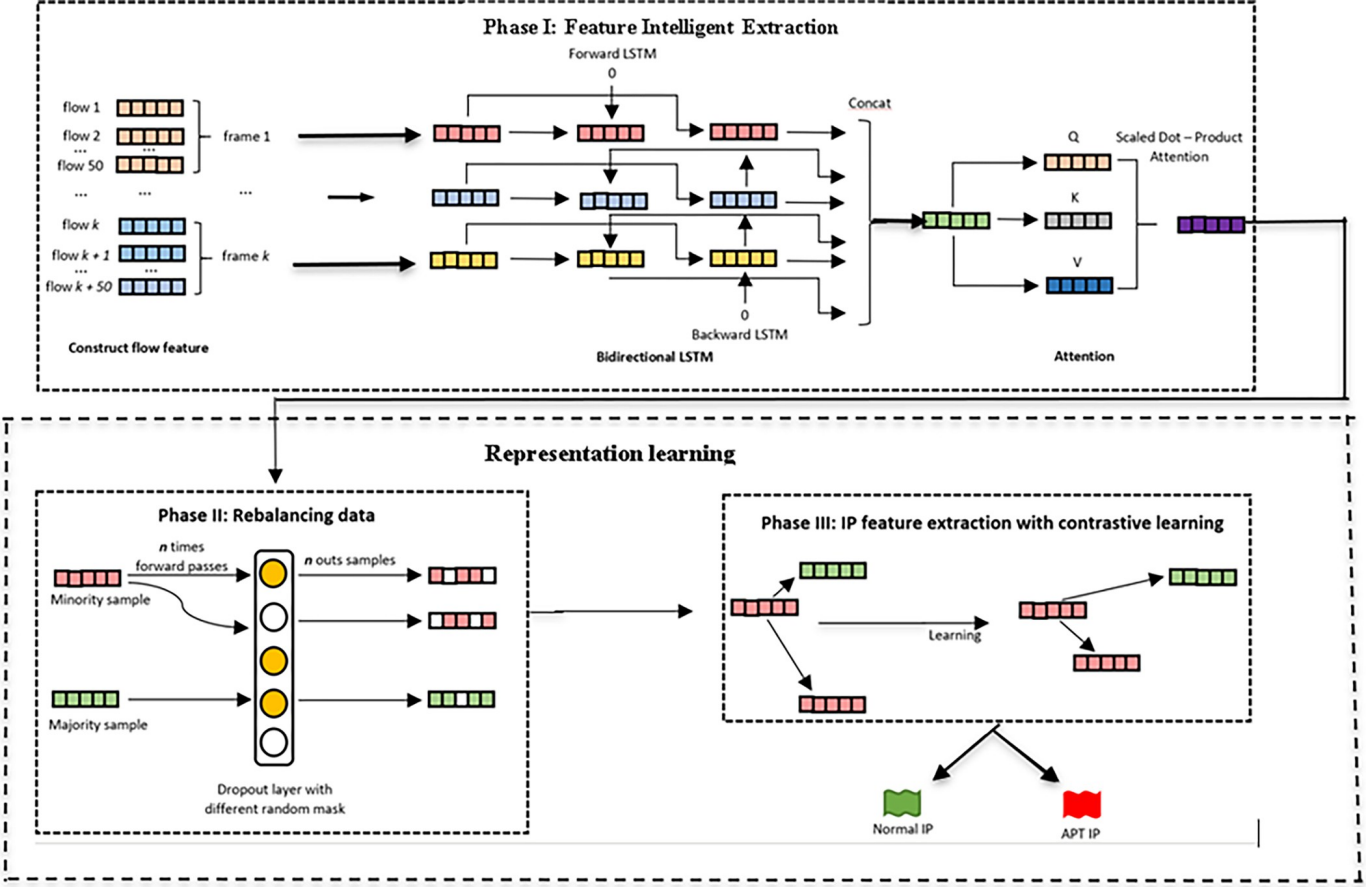
The flow of the FIERL model.

Fig 1 illustrates the flow of the FIERL model based on Feature Intelligent Extraction and Representation Learning for APT attack detection. The proposed model includes 2 main blocks with different tasks and processing functions as follows:

**i) Feature Intelligent Extraction Block** based on BiLSTM-Attention combined model perform 3 steps for synthesizing and extracting information of flows:

**Step 1: Building frames based on the flow network**: After flows are extracted, they are aggregated into frames of size 50x76. Where 50 is the number of flows in the frame and 76 is the number of features of each flow.

**Step 2: Extracting features based on frames**. At this step, the frames are fed into the BiLSTM network to extract and aggregate features of adjacent flows in the frame. Output is feature vectors with salient features of each frame after extracting from the flow network.

**Step 3: Aggregating and highlighting features by IP**: This step uses the Attention network to aggregate salient features of frames. Output is a vector representing each IP.

**ii) Representation Learning Block based on a model combining Dropout and contrastive learning**: There are 2 main blocks proposed in the Representation Learning Block as follows:

**Rebalancing Data Block** to reduce data imbalance when the number of normal IPs is too large in the dataset, making it easier for the model to learn and classify IPs. In the proposed model, the paper uses Dropout as an augmentation technique. When passing any vector through Dropout, the Dropout layer produces a vector similar to the original vector with some deactivated positions (assigned value 0). This paper uses the benefit of Dropout to add IPs that are labeled APT when passing their feature vectors through the Dropout network.

**iii) IP Feature Extraction with Contrastive Learning Block**: This block learns, recognizes, and classifies normal IPs and APT IPs based on feature vectors collected from the above two blocks. The purpose of the proposed use of Contrastive learning is to cluster IP feature vectors with the same label, "pull" similar data pairs together to learn each other’s higher-level features, and "push" away pairs of contrasting data. From there, the classification of IP labels becomes easier and more efficient.

### 3.2. Flow feature intelligent extraction

As described above, the model performs the function of analyzing, extracting, and synthesizing abnormal behaviors of APT IP with the combination of two networks: BiLSTM and Attention. Next, the paper will focus on clarifying each component.

#### 3.2.1. Data preprocessing

The CICFlowMeter tool [4,42] is suggested to extract flow networks in network traffic. It can analyze network traffic into flow networks and statistical features of flow networks. Specifically, CICFlowMeter analyzes each flow into 75 features. Based on the flows and 75 features as above, flows belonging to an IP are stacked into frames of size 100x75, where 100 is the number of flows in the frame and 75 is the number of features of each flow. However, the number of flows corresponding to each IP is different, so two special cases can happen: i) The number of flows exceeds 100: In this case, the flows are still stacked as k frames of size 100x75. Then the representative vector of the frame is the mean of these k frames; ii) The number of flows is less than 100: With IPs containing k flows (k < 100), (100 - k) zero vectors are padded so that the frame has a fixed size of 100x75.

#### 3.2.2. Aggregating and extracting flow features using BiLSTM

**a) Introduction to the BiLSTM network**

To extract flow features in the network traffic, the study [25] presented some combined deep learning network models such as CNN-LSTM, CNN-MLP, etc. However, in that study, the authors also explained some of the difficulties encountered by these networks in extracting features. However, when comparing and evaluating the effectiveness of these combined deep learning models with individual deep learning networks, the results show that the combined networks are much more effective than individual ones. From that, it can be seen that the idea of using combined deep learning networks to extract flow network features in network traffic is correct and reasonable. In this study, instead of using cumbersome combined deep learning networks, we propose to use the BiLSTM network for feature extraction. We take advantage of this network’s outstanding advantage, which is the ability to remember long distances and 2 dimensions, for attribute extraction. Accordingly, studies [43,44] introduced the BiLSTM model consisting of 2 parts: forward LSTM and backward LSTM. This allows the model to not only inherit the long-distance memory capacity of LSTM but also can remember 2-dimensional information. The two layers of LSTM generate two hidden states respectively, $h_{i}^{f}$ from forward LSTM and $h_{i}^{b}$ from backward LSTM. In which, $h_{i}^{f}$ integrates the forward information $h_{i}^{b}$ integrates the backward information. Determining the final state *h*_*i*_ by concatenating 2 states using Formula (1) below:

$$h_{i}=h_{i}^{f}||h_{i}^{b}$$ (1)

Where:

- *h*_*i*_ is the state at state *i* (contains information from both directions)
- || is a join operation

The study uses a BiLSTM model with 2 hidden layers for the purpose of extracting and synthesizing features of the frames built in the previous step.

**b) Flow feature extraction using BiLSTM**

Accordingly, after flow networks are processed into frames as in section 3.2.1, these frames are put into BiLSTM networks to extract and combine features of adjacent flows (see Fig 1). Next, the result of this process is a vector containing the salient features of the frames. This context vector represents the outstanding features corresponding to each original frame.

#### 3.2.3. Selecting and extracting IP features using attention

**a) Introduction to the Attention network**

To extract and highlight important information about IP based on a flow network, the study [24] proposed to use of an Inference network. The inference network is a relatively widely used network in the natural language processing field. However, we found that using an Inference network for flow network-based IP feature extraction has many disadvantages. Specifically, it is difficult to calculate and represent the relationship between frames in general and flow networks in particular based on Inference networks because flow networks operate independently and have very little information related to each other. To resolve the issues of the Inference network in the study [24], in this paper we will use the Attention network. Attention is one model of great concern in the field of deep learning [32]. It is first introduced by Dzmitry Bahdanau et al. [45] for the problem of machine translation. Jiachen [46] proposed applying Attention to the text classification task. Besides, Colin Raffel [47] proposed the Attention model to resolve the problem with strings of medium and large lengths.

In this paper, we use the Attention network to highlight important features and information of IP through the flow network [32]. The use of the Attention network helps the research model to automatically learn and determine which frames are important and contribute more to the context vector of each IP. The Attention vector that carries the score information of the context vectors is calculated as follows (2):

$$Attention(Q,K,V)=softmax\;\left(\frac{QK^{T}}{\sqrt{d^{k}}}\right)V$$ (2)

Where:

- *Q*, *K*, *V* are the weight matrices that need training model,
- coefficient *d*^*k*^ is the number of dimensions of the vector K.

**b) Extracting IP features using the Attention network**

At this phase, feature vectors of frames are passed through the Attention network to find and make stand out important features, rather than averaging like other classification methods. In particular, this process is as follows: First, after the BiLSTM model handles frame networks, all network’s hidden states are retained by flexibly combining processing blocks including *Q*, *K*, *V*, etc. Thus, the Attention network helps to create an output vector where the IP features are aggregated and made stand out by handling and evaluating the frame network. Obviously, there is a large difference between the output data of the BiLSTM model and that of the Attention network. Specifically, in the output vector of BiLSTM, features near the end are extracted and stored more than those at the front. In the output vector of Attention, the important features can appear anywhere because this vector contains features collected based on the entire data. From here it can be seen that with the support of the Attention network, the important features of IP based on the flow network are extracted and highlighted.

### 3.3. Representation learning for APT IP detection

In section 3.2 we detailed the process of collecting and building APT IP abnormal behavior. Next, instead of putting these APT IP behaviors in the classification process, we will process them by using the RL model. Contents 3.3.1 to 3.3.3 describe this process in detail.

#### 3.3.1. Rebalancing data

After obtaining the Attention vectors from the Flow Feature Extraction block, we noticed that there is a significant difference between the number of normal IPs and the number of APT IPs in the dataset. Specifically, the number of normal IPs is about 16 times more than the number of APT IPs. Therefore, if this problem is ignored, it will greatly affect the training process of the model because it learns too much information about normal IP as well as blurs the information of APT IP. To solve the data imbalance in the training process, we propose to use the Rebalancing data method after the Attention network. To achieve this requirement, recent studies and approaches used the synthetic minority over-sampling technique (SMOTE) to balance the number of samples in each different class (remove in the majority class and generate more neighboring vectors in the minority class). This traditional approach has many advantages and has been applied in many papers in different fields. In this paper, we propose to use the Dropout technique to generate data. The descriptions in a) and b) below shows the advantages of Dropout over Smote.

**a) Introduction to the SMOTE model**

SMOTE is a sampling method to increase the sample size of the minority group in case of sample imbalance, proposed by Chawla in 2002 [48]. To generate new samples, this algorithm computes random linear interpolation between several samples and their neighboring ones. After a certain number of artificial minority samples are generated, the data imbalance rate will increase, and the categorical completeness of the unbalanced dataset is improved. It works by varying the frequencies of different layers in the data. Specifically, SMOTE randomly deletes some examples to subsample the majority class and generates composite examples to subsample the minority class until all classes have the same frequency. Specifically, SMOTE subsamples the majority class (i.e. randomly deletes some examples) while supersampling the minority class (by generating composite examples) until all classes have the same frequency. In the case of APT attack prediction, the minority class is an APT malware sample. SMOTE is effective in domains with unbalanced datasets. The execution procedure of the SMOTE algorithm is as follows [48]:

- For each minority set *x*_*i*_ (*i* = 1, 2, …, *n*), calculate its distance to other samples in the minority sample according to certain rules to obtain k nearest neighbor samples of it.
- According to the over-sampled gain, m nearest neighbor random samples will be as a subset of k nearest neighbor samples, of each set of *x*_*i*_ chosen and denoted by *x*_*ij*_ (*j* = 1, 2, …, *m*), then a built-up minority sample pij calculated using the following equation:

$$p_{ij}=x_{i}+rand(0,1)\times (x_{ij}-x_{i})$$ (3)

Where, *rand*(0,1) is a uniformly distributed random number in the interval [0,1]. The operation cycle of the algorithm will stop until the data merge reaches a certain imbalance ratio.

Comment: From the process and operation of Smote, it can be seen that this is a method of generating neighbor data based on the distance of samples with the same label in space. This approach has difficulties in applying to the APT attack datasets because the characteristics of APT attacks are much different from normal behaviors. Therefore, theoretically, we think that the Smote technique is effective for the APT attack detection model. However, it is not the best technique for creating APT attack detection models.

**b) Proposal to use Dropout technique**

To overcome the disadvantages of SMOTE in this paper, we propose to use Dropout. Dropout was first launched in 2014 [49] and is commonly used as a method to solve the problem of overfit learning during training by randomly disabling some connections from the previous layer (output is 0). The study [50] presented some concepts and definitions of Dropout. The research [51] used Dropout on a representation vector *x* = (*x*_1_, *x*_2_,…*x*_*d*_), each component *x*_*k*_ (*k* = 1,2,…*d*) becomes:

$$\hat{x_{k}}=a_{k}.x_{k}$$

Where: *a*_*k*_~*P* is a random variable with the Bernoulli distribution:

$$P(a_{k})=\left\{ \begin{array}{l} 1-p,\;a_{k}=0\\ p,\;a_{k}=1 \end{array}\right.$$

Based on this idea, the study puts the Attention vector through the dropout function n times to create additionally n more samples $\hat{x_{k}}$ have the same class with the same features and information as the initial context vector x_k. With this approach, the generated sample vectors still have neighboring properties with original feature vectors even though they are not generated by spatial distance. Detailed representations of the contribution of the Dropout method are described later in the study.

#### 3.3.2. Representation learning method

The trend of using Representation learning for classification problems is being applied a lot nowadays. In the study [52], the Representation learning method was used for the task of classifying source code vulnerabilities. Specifically, in their research, the authors proposed the Triplet Loss technique to optimize the source code vulnerability classification process. In this paper, we propose a new Representation learning method based on the Contrastive learning technique. Next, we will go into a detailed description of the technical characteristics of these two methods.

**a) Introduction to Triplet Loss**

Triplet Loss was first introduced at [53] in 2015 and is one of the most prevalent loss functions. Triplet Loss encourages that different pairs be at least a certain distance away from any similar pair. The loss value is calculated by the following formula:

L=max(d(a,p)−d(a,n)+m,0) (4)

Where:

- *p*: is the sample with the same label as *a*
- *n*: is the sample with the different label as *a*
- *d*: is the distance function
- *m*: is the margin value to set negative samples apart

**b) Proposal to use Contrastive learning**

Contrastive learning has been applied to many problems both in Computer Vision [54] and NLP [55], showing remarkable efficiency, beating many SOTA models. The main idea of Contrastive learning is to find pairs of similar and contrasting data features in a dataset. From there, pairs of similar data can be "pulled" together to learn higher-level features of each other, and pairs of contrasting data can be "pushed" away. To do this, we use similarity metrics to calculate the distance between the embedding vectors representing the data points. For example, we already have an original data point called anchor, then we can use different augmentation techniques to get one more variation from the original anchor or consider data points with the same label in the dataset as positive samples, and consider the rest of the batch/dataset or other data points with a different label as negative samples. Then the model is trained to be able to classify positive samples and negative samples from a data cluster. In this paper, we propose to use Contrastive learning for the task of learning important features of feature vectors. The operation principle of the Contrastive learning method is as follows:

Suppose we have a set of data points $D=\{(x_{i},x_{i}^{+},x_{i}^{-})\}$. In which, *x*_*i*_ and $x_{i}^{+}$ are 2 data points that are similar or have the same label, *x*_*i*_ and $x_{i}^{-}$ are 2 data points that contrast or have different labels. Call $h_{i},h_{i}^{+},h_{i}^{-}$ are the representative vectors of $x_{i},x_{i}^{+},x_{i}^{-}$ respectively, then the training objective of a mini-batch *N* is defined as Formula (5):

$$L_{cl}=\sum_{i=1}^{n}L_{i}$$ (5)

With *L*_*i*_ is defined according to Formula (6) as follows:

$$L_{i}=-\frac{1}{N_{y_{i}}}.\sum_{k=1}^{N}1_{i\neq k}.1_{y_{i}=y_{k}}.\log\frac{e^{\frac{sim(h_{i},h_{k})}{\tau }}}{\sum_{j=1}^{N}1_{i\neq j}{.1_{y_{i}\neq y_{j}}e}^{\frac{sim(h_{i},h_{j})}{\tau }}}$$ (6)

Where: *τ* is a positive constant temperature hyperparameter,

- *sim* (*h*_1_, *h*_2_) is cosine similarity $\frac{h_{1}^{T}h_{2}}{\left|\left|h_{1}\right||.|\left|h_{2}\right|\right|}$
- *1*_*B*_ = 1 when *B* is true, otherwise 1_*B*_ = 0,
- *N*_*y*_ is the total number of samples in the mini-batch with the same label *y*,
- *i* is the index of the example in the mini-batch,
- *k* is the index of other examples in the mini-batch with the same label with the example index *i* or $x_{k}=x_{i}^{+}$,
- *j* is the index of other examples example has index *i* in mini-batch.
- *h*_*k*_ is the representative vector of $x_{i}^{+}$.

#### 3.3.3. APT IP classification

With 2 techniques Triplet Loss and contrastive learning described in Section 3.3.2, we will conduct APT IP classification based on these techniques. The study [52] proposed combining MLP with Triplet Loss and also brought a relatively good effect. However, in our research, we propose to use Cross-Entropy Loss. The mathematical process of the two models is described in (a) and (b) as follows:

**a) The classification model combining MLP and Triplet Losses**

Embedding APT IP samples and normal IP samples in phase 2 tends to express a large degree of overlap in the object space. This lack of separation makes it difficult for the classification process to distinguish between APT samples and normal samples. To improve prediction performance, we use a model that can predict features from the original high overlap space into another space that provides better separation between APT samples and normal samples. For this, we use an MLP network designed to transform the input feature vector *(x*_*g*_*)* into a latent representation denoted by *h(x*_*g*_*)*. To optimize the separation between APT samples and normal samples, we use a combination of three loss functions (Triplet Losses) as the loss function of the classification model. Triplet losses consist of 3 single loss functions: i) Cross Entropy (ℒ_*CE*_); ii) Projection loss or Triplet Loss (ℒ_*p*_); and iii) L2 Regularization (ℒ_*reg*_). The mathematical formula below shows the relationship of Triplet losses.


$$L_{trp}=L_{CE}+\alpha *L_{p}+\beta *L_{reg}$$ (7)

Where: α and β are two hyperparameters representing the contribution of ℒ_*p*_ and ℒ_*reg*_. The operating principles of each component in Triplet losses are as follows:

First, Cross Entropy is used to calculate the error in the classification result. The value of the Cross Entropy loss function increases when the probability that the predicted label differs from the actual label. It is expressed through the formula:

$$L_{CE}=-\sum ŷ.log(y)+(1-ŷ).log(1-y)$$ (8)

Which, *y* is the actual label and ŷ is the predicted label.

Next, Triplet Loss is used to quantify how well the performance can separate vulnerable samples from non-vulnerable samples. A representation is said to be useful if all vulnerable samples in the potential space are close to each other and at the same time it will be far away from all non-vulnerable samples, i.e. samples from the same class will be very close together and samples from different classes will be far apart. Accordingly, the formula of the Triplet Loss function will be determined according to the following:

$$L_{p}=|D(h(x_{g}),h(x_{same}))-D(h(x_{g}),h(x_{diff}))+\gamma |$$ (9)

Which, *h*(*x*_*same*_)) is the latent representation of an instance belonging to the same class as *x*_*g*_ and *h*(*x*_*diff*_)) is the latent representation of an instance of a class other than *x*_*g*_. *γ* is a hyperparameter used to determine the minimum separation boundary. Finally, D(·) represents the cosine distance between the two vectors and is given by:

$$D(v_{1},v_{2})=1-\left|\frac{v_{1}.v_{2}}{\left\|v_{1}\|*\|v_{2}\right\|}\right|$$ (10)

If the distance between two samples belonging to the same class is large ($D(h(x_{g}),h(x_{same}))$ are large) or if the distance between two samples of different classes is small ($D(h(x_{g}),h(x_{same}))$ are small), then ℒ_*p*_ will represent the opposite relationship between different classes.

The last is L2 Regularization with the symbol ℒ_*reg*_, this function is used to limit the magnitude of the latent representation (*h*(*x*_*g*_)). We find that, over several iterations, the latent representation *h*(*x*_*g*_) of input *x*_*g*_ tends to increase in size arbitrarily. Such an arbitrary increase of *h*(*x*_*g*_) prevents the model from converging. Therefore, we use the loss function ℒ_*reg*_ to deal with latent representations *h*(*x*_*g*_) of larger magnitude. The loss function ℒ_*reg*_ is described by the mathematical formula below:

$$L_{reg}=\|h(x_{g})\|+\|h(x_{same})\|+\|h(x_{diff})\|$$ (11)

**b) Proposing an APT IP classification model using contrastive learning and Cross-Entropy Loss**

After the data is balanced by the Dropout method and clustered according to the same characteristics by contrastive learning, in this phase we proceed to classify behaviors of APT IPs to detect normal IPs and APT IPs. We propose to use one Fully Connected Layer after the Contrastive Learning block with Cross-Entropy Loss. The Cross-Entropy Loss function is expressed through the following formula:

$$L_{ce}=-\frac{1}{N}\sum_{k=1}^{N}y_{i}.log(\sigma (h_{i}))+(1-y_{i}).log(1-\sigma (h_{i}))$$ (12)

Where:

- *h*_*i*_ is the representation vector of *x*_*i*_,
- *σ*(*x*) is sigmoid function.

Note that during this process, only parameters of the last classification layer are tuned, all parameters in the previous representation block are frozen.

## 4. Experiment and evaluation

### 4.1. Experimental dataset

APT attack data (positive label): use 29 real botnet traffic files from the Malware Capture CTU-13 dataset. These botnet traffics were captured from 6 APT malware types including Andromeda, Colbalt, Cridex, Dridex, Emotet, and Gh0stRAT [56].

Normal data (negative label): are captured on July 30, 2019, from the E-Government server of Soc Trang province [57] in the scientific research project No KC.01.05/16-20 of the Ministry of Science and Technology of Vietnam.

Table 1 presents the statistical information of experiment data collected and used in this paper. During the experiment, the data set is divided into two subsets, which are the training subset accounting for 80%, and the testing subset accounting for the remaining 20%.

**Table 1:** Details of the experimental data.

N°	Type	Total	APT	Normal
1	Flows	8.543.362	19.025	8.524.337
2	IP	157.126	7.375	149.751

### 4.2. Performance evaluation metrics

Four main metrics are used to evaluate the model during the experiments, including Accuracy, Precision, Recall, and F1-score. The general formula for these four measures is as follows.


$$Accuracy=\frac{TP+TN}{TP+TN+FP+FN}\times 100\%$$ (13)

$$Precision=\frac{TP}{TP+FP}\times 100\%$$ (14)

$$Recall=\frac{TP}{TP+FN}\times 100\%$$ (15)

$$F1=\frac{2\times Precision\times Recall}{Precision+Recall}$$ (16)

Where, TP is true positive; FN is false negative; TN is true negative; FP is false positive.

### 4.3. Evaluation scenarios

Some experimental scenarios in the paper are as follows:

- Scenario 1: How effective is the proposed approach in the paper? To answer this question, we refine some parameters in the proposed model to see the effectiveness of the model in the APT IP detection task.
- Scenario 2: Why use the FIERL model? To answer this question, we replace the FIE and RL models with some other models. Specifically, we in turn replace the FIE model with CNN, LSTM, and MLP-Inference networks. For the RL model, we experiment with replacing Dropout with Smote, replacing contrastive learning with Triplet loss, and finally replacing the whole RL model with some supervised machine learning algorithms.
- Scenario 3: Comparing the FIERL model with some other studies and approaches. In this scenario, we experiment with some other approaches and studies on APT attack detection on our experimental dataset.

### 4.4. Some experimental results

#### 4.4.1. Experimental results of scenario 1

As is known, in this paper we propose a model with a combination of 4 different techniques: BiLSTM, Attention, Dropout, and Contrastive learning. Each model has different effects on the accuracy of the classification. However, changing the parameters of all 4 techniques at the same time is cumbersome and difficult. Therefore, we propose to evaluate each model individually based on 2 main combined models, FIE and RL. Tables 2 and 3 below show the experimental results of detecting APT attacks according to the approach of the paper.

**Table 2:** Experimental results of detecting APT attack when the FIE model’s parameters are changed and parameters *τ* and *α* of RL model are fixed at 0.1 and 0.1 respectively.

BiLSTM + Attention		IP classification performance			
**BiLSTM nodes**	Attention nodes				
		Acc	Pre	Rec	F1
**128–128**	128	0.98	0.81	0.83	0.82
**128–256**	256	**0.99**	**0.84**	**0.89**	**0.87**
**256–256**	256	0.99	0.83	0.86	0.85

**Table 3:** Experimental results of detecting APT attack when the RL model’s parameters are changed and the FIE model’s parameters are fixed.

Representation learning	*τ*	Dropout											
		*α*											
		**0.1**				0.2				0.3			
		Acc	Pre	Rec	F1	Acc	Pre	Rec	F1	Acc	Pre	Rec	F1
	0.05	0.98	0.80	0.85	0.82	0.98	0.79	0.84	0.81	0.97	0.77	0.81	0.79
	**0.1**	**0.99**	0.84	**0.89**	**0.87**	0.99	**0.85**	0.84	0.84	0.98	0.81	0.83	0.82
	0.2	0.98	0.84	0.87	0.85	0.98	0.82	0.85	0.83	0.98	0.79	0.83	0.81

To evaluate the influence of BiLSTM and Attention models in the process of extracting flow information, we change the number of nodes of the layers in turn. The results in Table 2 show that when changing the parameters of the BiLSTM and Attention models, the experimental results also change. However, this change is not too large and not even. Specifically, the best and lowest Accuracy results differ by about 1%. Similarly, for the Precision measure, this difference is about 3%. Finally, with the Recall and F1-score measures, these differences are 6% and 5%, respectively. On the other hand, it can be seen that, when the number of nodes in the BiLSTM layer is 128-256 and in the Attention layer is 256, the model gives the best results in which Accuracy, Recall, and Precision values are superior to remaining experiments. When the number of nodes is too small for the model to learn and extract important features in the flow network, the model doesn’t achieve the best performance. In contrast, when the number of nodes is large, the model focuses too much on the data points in the training set (overfitting phenomenon), which makes the classification result significantly reduces (Recall decreases by about 3% compared to the best model). This experimental result shows that in applying deep learning models and algorithms to the problem of detecting network attacks in general and APT attacks in particular, not that choosing the higher the parameters and the larger the model, the better the effect. The model must fit the characteristics of the experimental data. Table 3 below shows the APT attack detection results when only the RL model’s parameters are changed and the FIE model’s parameters are fixed according to the best results as shown in Table 2.

In Table 3, we fine-tune 2 parameters *α* and *τ*. In which *α* is the dropout probability, the larger *α* is, the more values in the representation vector are deactivated; *τ* is the temperature parameter that helps control the punishment with negative samples. Research [58] shows that the smaller *τ* is, the more contrastive loss tends to focus on regions of great similarity. However when *τ* is too small, the contrastive loss may focus on only a few samples that are most similar to the sample being trained, then this greatly affects the quality of the model.

Looking at Table 3, the model gives the best results when *τ* = 0.1 and *α* = 0.1 with the APT attack detection rate is up to 99%. It is easy to see that when the Dropout rate *α* is small, the data points generated from the Dropout layer are more similar to the original data points than the data points generated with the bigger Dropout rate *α*. This makes the model more compatible with a small Dropout rate *α*, when *α* gradually increases from 0.1 to 0.2, 0.3, the result decreases significantly. With temperature parameter *τ*, obviously with *τ* neither too small nor too large, the model has markedly better results in the APT attack classification task. When *τ* = 0.05 (too small), the model only focuses on a small area of similar data points, reducing Precision and Recall by about 4% compared to *τ* = 0.1. On the contrary, when *τ* = 0.2 (too large), the model is too affected by the distant data points that may not really be similar to the data point being considered, which directly affects the training results when Recall is also reduced by 4% compared to *τ* = 0.1.

**Fig 2:**
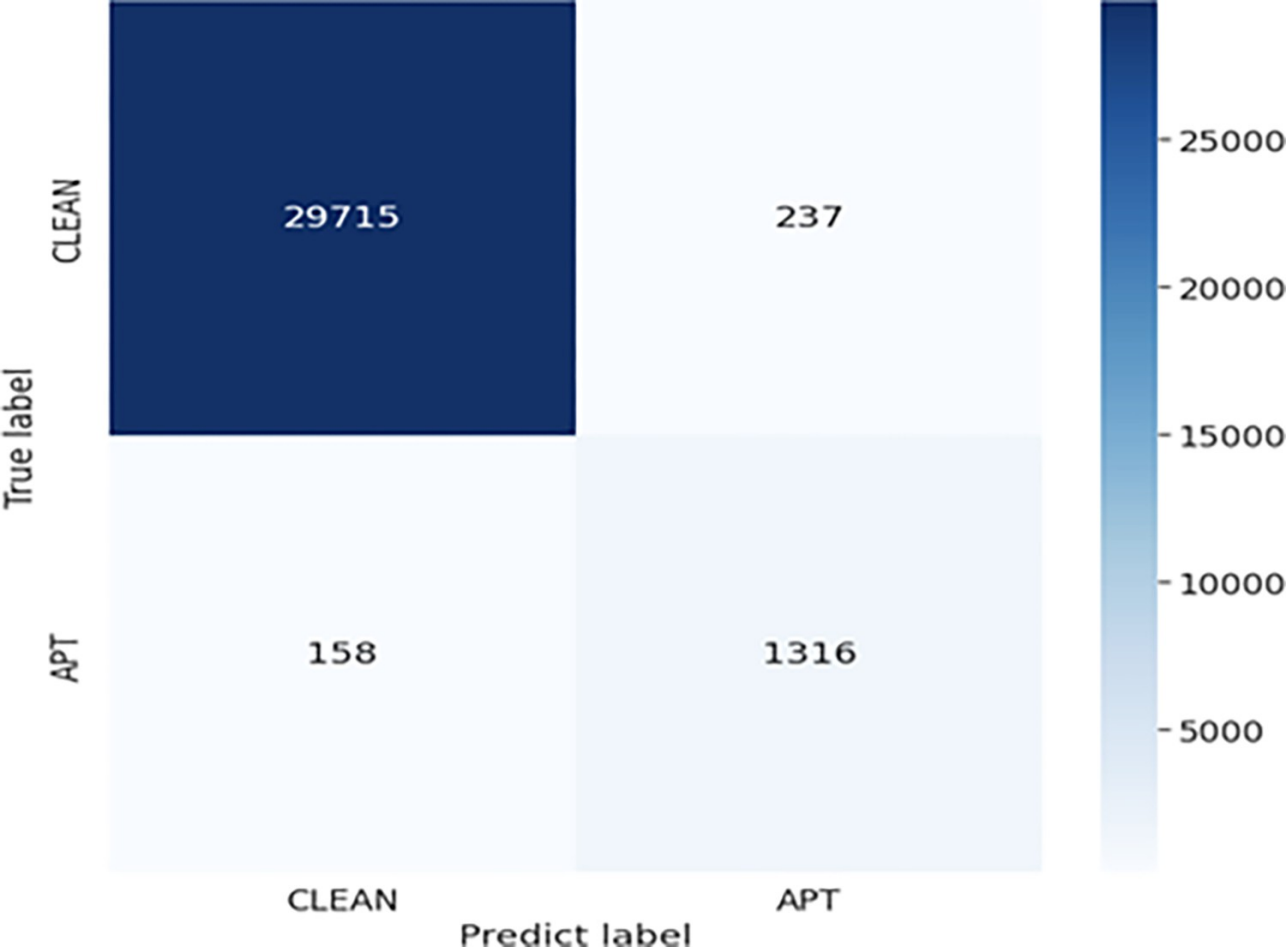
Confusion matrix result of the FIERL model with the best parameters.

Combining Tables 2 and 3, we select the model FIERL with the best parameters *τ* = 0.1, *α* = 0.1, BiLSTM is [128, 256], and Attention layer is 256. Fig 2 shows the best Confusion matrix results of applying the FIERL model for APT attack detection.

The confusion matrix result of the model FIERL shows that the model correctly predicts 1,316 APT IPs out of a total of 1,474 APT IPs (89.28%) and has a false prediction rate of only 10.72%. Besides, for the prediction results of normal IPs, the FIERL model also gives very good prediction results when the false prediction rate of normal IPs is only 0.79%. Looking at the correct prediction rate of APT IPs as well as normal IPs, it can be seen that the APT IP prediction model is not effective. However, from a data perspective, this result is very good for APT detection models in reality. In particular, in the experimental dataset described in Table 2, the number of normal IPs accounts for a very large number of the total experimental IPs. The number of normal IPs is 20 times the number of APT IPs Therefore, with such a difference, the FIERL model still achieves the results shown in Fig 2, it is clear that the FIERL model has worked very well. This demonstrates the Feature Intelligent Extraction and Representation Learning techniques proposed in the paper have shown their effectiveness. This demonstrates that 2 techniques proposed in the paper, Feature Intelligent Extraction and Representation learning, have shown their effectiveness.

#### 4.4.2. Experimental results of scenario 2

The experimental results in Tables 2 and 3, and Fig 2 have shown the effectiveness of the model FIERL. In this scenario, we compare the FIERL model with some other models. Specifically, the paper replaces FIE with some other deep learning networks such as CNN, LSTM, and MLP. These experimental results are presented in section (a) below. Similarly, to evaluate the effectiveness of the RL method, we proceed to replace this method with some other combined model. Section (b) shows some results of replacement models: replace Dropout with Smote, replace Contrastive Learning with Triplet loss, and finally replace the whole RL model with some supervised machine learning algorithms.

**a) Experimental results when replacing the FIE model with some deep learning networks**

**Fig 3:**
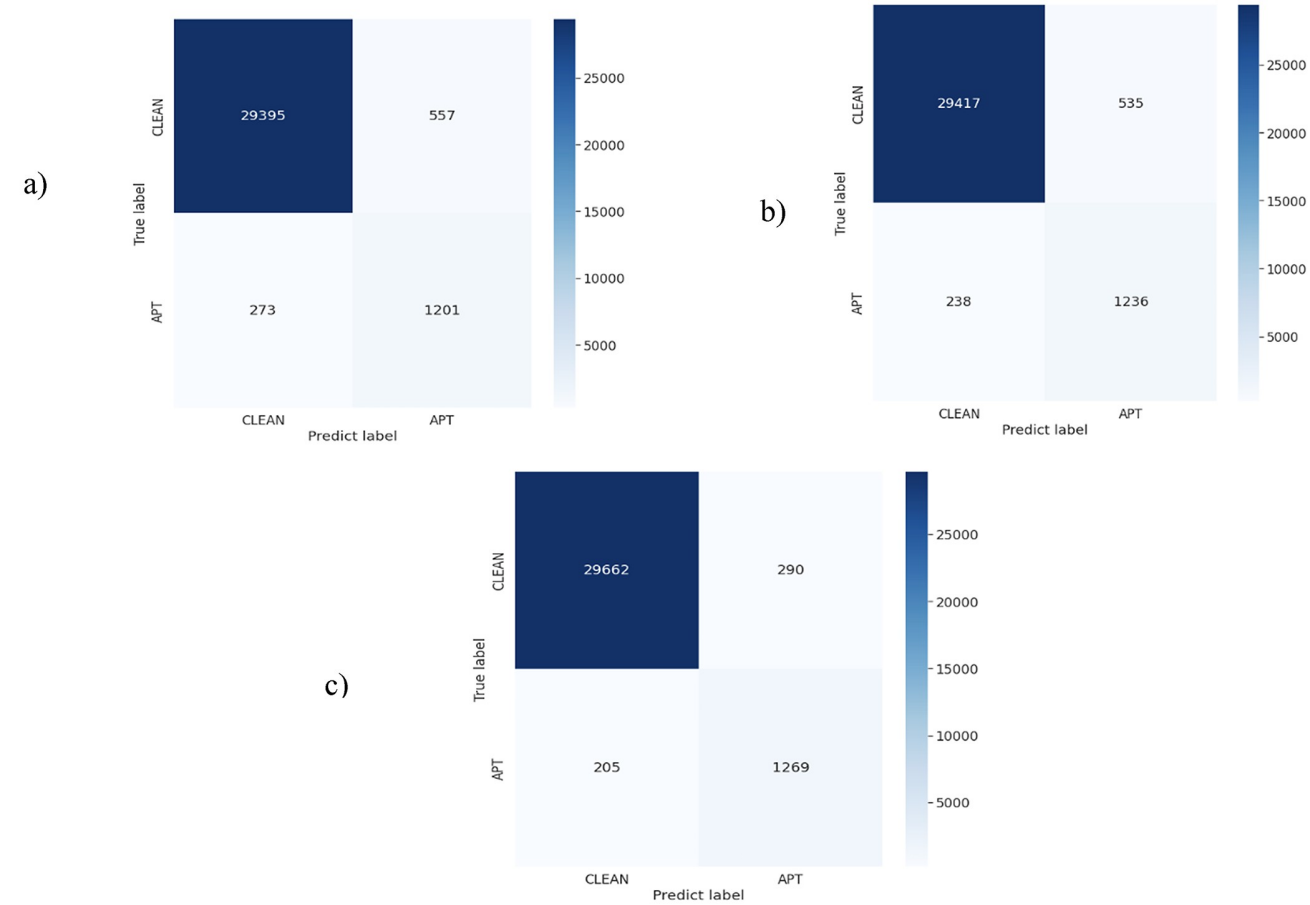
Confusion matrix results of models using deep learning networks to replace the FIE model. In which (a): CNN; (b): LSTM; (c): MLP-Inference.

The experimental results in Table 4 show that when replacing the FIE model, experimental results significantly reduce. Specifically, when only using CNN to extract flow features, this model gives very low results. These results are lower than the results of the proposed model in the paper (shown in Table 3) by nearly 10% on all measures. When using the LSTM network to extract features, the result is better than using the CNN network, because the LSTM network can remember the features, so it extracts better flow features and helps the model to synthesize more IP features. However, it is still very poor compared to our proposed FIERL model. Finally, using the MLP-Inference combined model to extract flow features gives better performance than the LSTM and CNN models. The reason is that the MLP network helps to synthesize flow features, then with the support of the Inference network, relationships of flow networks are extracted and clarified. From here, normal and APT flow networks are clearly distinguished, thereby helping the Dropout-Contrastive network to promote its effectiveness in aggregating and classifying APT IPs. Fig 3 below describes the Confusion matrix results of deep learning networks used to replace the FIE model. The Confusion matrix results shown in Fig 3 again represent that deep learning models such as CNN, LSTM or MLP-Inference cannot give good performance for the task of classifying normal IPs and APT IPs. Comparing the results shown in Figs 2 and 3, it can be seen that there is a huge difference between the FIERL model and the other models. Specifically, when using CNN to replace FIE, this model incorrectly predicts up to 273 APT IPs. This false prediction result is higher than the FIERL model to 115 APT IPs. This difference in the model using LSTM is 80 APT IPs. Finally, the MLP-Inference combined network also incorrectly predicts 205 APT IPs, which is 47 APT IPs higher than our proposed FIERL model. From this, it can be concluded that using some deep learning models to replace the FIE model in the task of aggregating and extracting IP features is not effective. Therefore, the proposal to use the FIE model in this paper not only has scientific significance but also has great practical significance.

**Table 4:** Experimental results when replacing the FIE model with some deep learning networks.

Model	Acc	Pre	Recall	F1
CNN - Dropout-Contrastive	0.98	0.68	0.81	0.74
LSTM – Dropout-Contrastive	0.98	0.70	0.84	0.76
MLP - Inference -Dropout-Contrastive	**0.98**	**0.81**	**0.86**	**0.84**

**b) Experimental results and evaluations for the RL model**

In scenario 1, we have presented some experimental results to prove the effectiveness of the proposed model in the APT IP classification task. For this scenario, our purpose is to answer the following research question (RQ):

RQ: What is the role and importance of each Dropout and Contrastive Loss component in the task of optimizing APT attack detection? Why combine Dropout with contrastive loss? Why not other combined methods? Are other combined models more effective than the model combining Dropout and Contrastive Loss? To answer this RQ (ARQ), we conduct the following experiments:

ARQ1: Evaluating the role and effectiveness of the Dropout method in the task of rebalancing data. To accomplish this task, we use the Smote method to replace the Dropout method in the rebalancing data task. The experimental model is a combination of the FIE model with Smote and Contrastive Loss.

ARQ2: Evaluating the role and effectiveness of Contrastive Loss in the Representation Learning task by replacing the Contrastive Loss method with the Triplet Loss method. The experimental model is a combination of the FIE model with Dropout and Triplet Loss.

ARQ3: Don’t use Contrastive Learning. Specifically, after the data is processed by the Dropout method, it is classified by traditional machine learning and deep learning methods such as MLP and RF. The experimental model is a combination of the FIE model with MLP or RF.

Table 5 below presents the results of the proposed models in ARQ1, ARQ2, and ARQ3 under scenario 2.

**Table 5:** Experimental results when replacing components in the RL model.

ARQ	Approach	Acc	Pre	Rec	F1
ARQ1	**Smote and Contrastive Loss**	**0.99**	**0.84**	**0.85**	**0.85**
ARQ2	**Dropout and Triplet Loss**	0.98	0.82	0.81	0.81
ARQ3	FIE model with MLP	0.96	0.75	0.77	0.76
	FIE model with RF	0.96	0.73	0.77	0.75

Based on the experimental results in Tables 2, 3 and 5, we have the following comments:

**Comment #1 for ARQ1**: Comparing the results of Tables 2 and 3 with Table 5, it can be seen that: The Dropout-Contrastive Loss combined model proposed by us yields better results than the Smote-Contrastive Loss combined model. This shows that the Dropout method is more effective than the Smote method in rebalancing data in the embedding space. So what is the reason that makes the Dropout method more effective than the Smote method? Fig 3 depicts the distribution of the dataset when using the Smote and Dropout methods to rebalance data.

**Fig 4:**
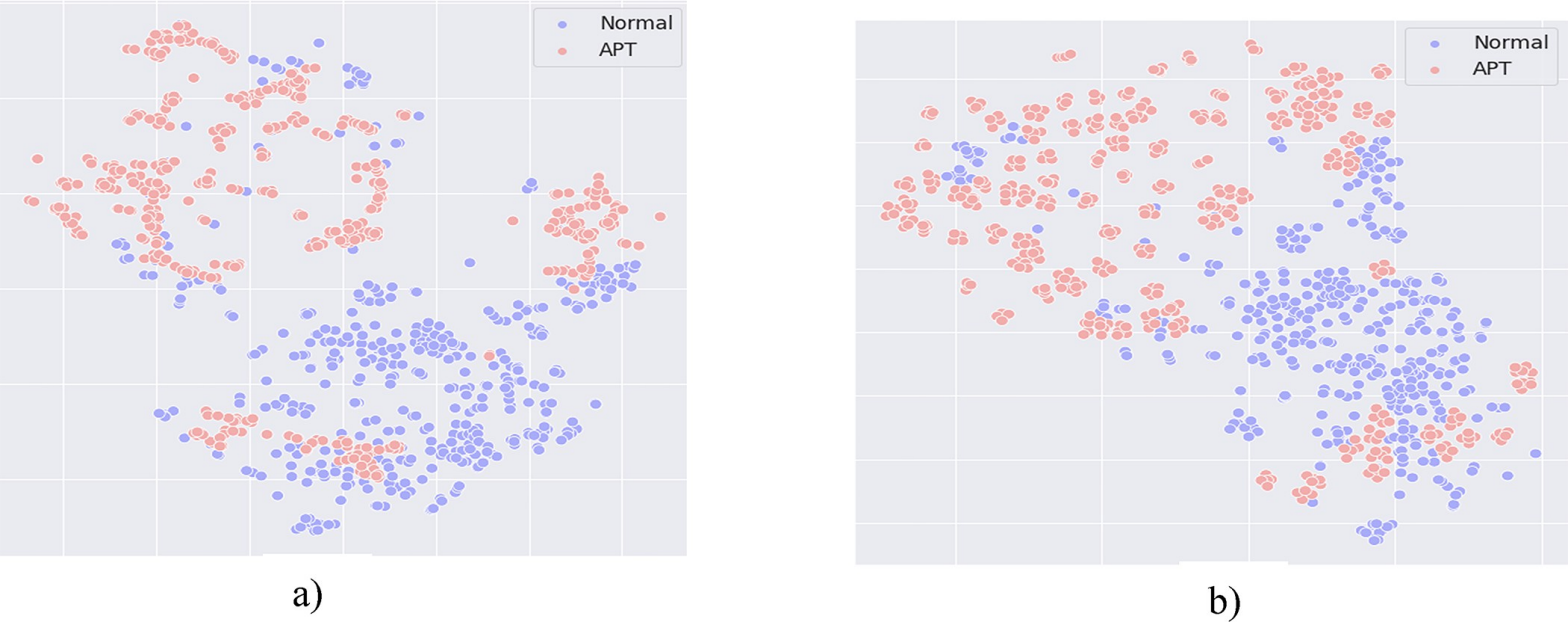
Comparing the difference in data distribution of the Smote and Dropout methods. Where: (a): The data distribution of the Smote algorithm; (b): The data distribution of the Dropout algorithm.

Fig 4 shows that the Smote method has succeeded in adding new data points to help balance the distribution between two labels in the dataset. Comparing the data distribution in Fig 4(A) (using the SMOTE method) and Fig 4(B) (using the Dropout method), it is clear that the Dropout method has many advantages over the SMOTE method in the task of adding new data points from the original dataset. To generate a new data point, SMOTE uses interpolation between two or more neighboring data points in the representation space. From Fig 4, it is easy to see that SMOTE is quite sensitive to noisy data points, the newly generated data points are distributed unfocused and have a large variance, which changes the distribution of the original dataset. The Dropout method overcomes this shortcoming of Smote by using only one data point to generate similar data points. Although the generated data points don’t have a relationship between the proximity distances in the representation space like Smote, observing Fig 4(B) clearly shows that these data points still have similarities and neighbors in the representation space. In addition, Dropout is less sensitive to noise because the generated data points can only be located in neighboring the original data point (when the Dropout rate is not too large), this helps the dataset after balancing to keep the same distribution as in the original dataset. Furthermore, the Dropout method also uses fewer resources and has a much lower computation time than the Smote method. The reason is that to find out the neighboring data points in the Smote method, it must run a clustering algorithm such as KNN and then calculate to generate new data points. Besides, it is easy to see a serious problem when using Smote with large datasets: clustering models take a lot of time to find the closest neighbors to the original data point. In contrast, with Dropout, we simply combine the representation vector of the original data point with a sparse vector to generate a new data point. This further proves the suitability and advantages of using Dropout for the rebalancing data method.

**Fig 5:**
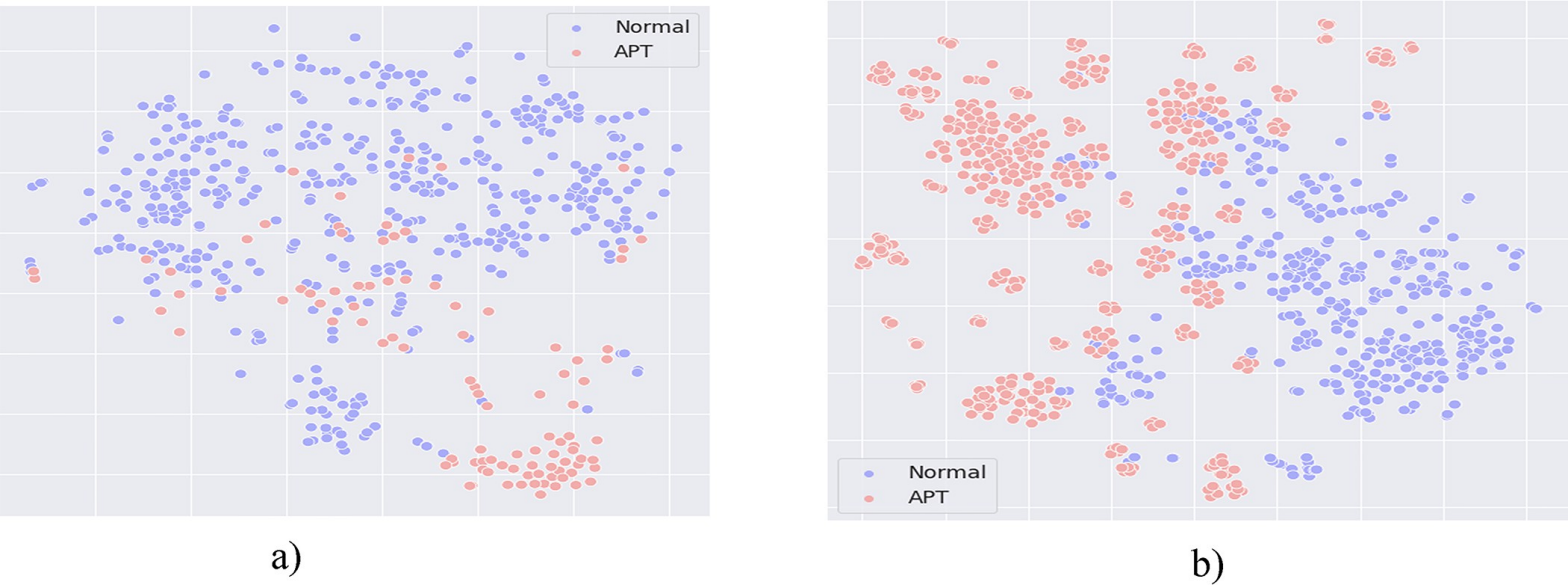
(a): The data distribution when using a combination of Dropout and Triplet Loss; (b): The data distribution when using a combination of Dropout and Contrastive Learning.

**Comment #2 for ARQ2**: The Dropout-Contrastive Loss combined model proposed by us brings much better performance than the Dropout-Triplet Loss combined model. This efficiency is most clearly demonstrated in the ability to accurately detect APT IPs when our proposed method brings about 1%-7% higher efficiency. Therefore, it can be concluded that Contrastive loss has better performance than Triplet loss in the task of training the model to give representation vectors Fig 5 below shows the data distribution after being processed by the Dropout-Triplet loss combined model.

From Fig 5 it can be seen that: Due to the nature of Triplet loss, the model can only train through a pair of positive and negative data. However, the rebalancing data method by using Dropout that we propose can generate more than one pair. Therefore, with data that has a lot of positive samples generated, Triplet Loss doesn’t take full advantage of the rebalancing data methods. On the contrary, using Contrastive Loss successfully overcomes this limitation of Triplet Loss when it helps the model to train with data that has many positive samples generated, taking full advantage of rebalancing data methods.

**Comment #3 for ARQ3**: For models that don’t use representation learning, it is clear that their effectiveness is very low. Accordingly, if only using the Dropout method to generate data for the training model and without using the Contrastive loss method to optimize the classification process, it won’t effective. From Fig 4 in scenario 1 of the paper, we have presented the distribution of data after using the data rebalancing technique. From Fig 3, it can be understood that the cause of this problem is that the vector representation of APT IP and normal IP in the embedding space is still fragmented and not centrally distributed. This shows that classification methods such as MLP or RF are not capable of identifying and classifying normal IPs and APT IPs.

**Fig 6:**
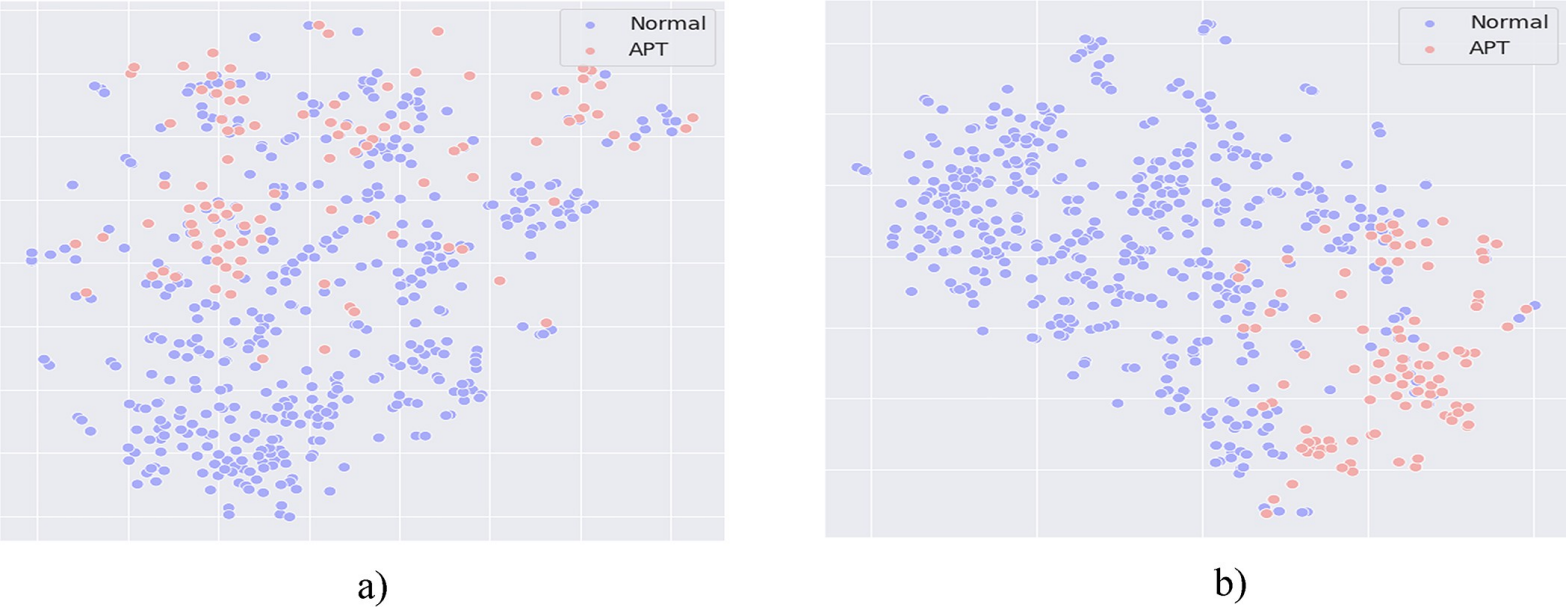
Comparing the data distribution of the method without using representation learning (a) and using representation learning (b).

Comparing the distribution results in Fig 6, it is clear that our proposed method with the combination of Dropout and Contrastive loss classifies clusters containing normal and APT labels better than not using representation learning. The data points in Fig 6(A) are still discretely distributed, the APT labels and normal labels are not separate, reducing the classification ability of the model. Observing Fig 6(B), it is easy to see that not only the data points of the APT label are grouped and concentrated, but the data points belonging to the normal label are also distributed into neighboring small clusters, which significantly improves the classification results of the model. From here, it can be seen the success of combining the two methods: Dropout and Contrastive loss. Finally, comparing the experimental results in Table 5 with Tables 2 and 3, it can be seen that the model when not using the representation learning method has the worst performance on all measures.

#### 4.4.3. Experimental results of scenario 3

Scenarios 1 and 2 have respectively demonstrated the effectiveness of the proposed model as well as the role of each FIE and RL component in the task of synthesizing and classifying APT IP. For this scenario, we conduct experiments for some approaches on the dataset used in the paper. Specifically, the study evaluates some combined deep learning networks: CNN-LSTM [25], BiLSTM-GCN [4], and MLP-Inference-GCN [24]. Table 6 below shows the best experimental results of these approaches.

**Table 6:** Experimental results of scenario 3.

Approach	Accuracy	Precision	Recal	F1
**Our approach**	**0.99**	**0.84**	**0.89**	**0.87**
BiLSTM-GCN [4]	0.97	0.73	0.62	0.67
CNN- LSTM [25]	0.96	0.65	0.40	0.49
MLP-Inference-GCN [24]	**0.99**	**0.86**	0.84	0.85

From the experimental results in Table 6, it can be seen that the results of the proposed method in this study are much higher than other approaches on the same dataset. Accordingly, when compared with the study [4], our study is higher from 2% to 27% on all measures. In particular, the Recall of the study [25] is lower than our proposed model by nearly 50%. In general, in all other approaches, our research direction is higher from 5% to 49% according to the Recal measure. The remaining results are also about 10% higher. Although the research [24] has a higher Precision than our study by about 2%, it has a lower Recall than our approach by about 5%.

## 5. Conclusion and future development direction

With 3 proposed experimental scenarios in the paper, we have proven the outstanding effectiveness of the proposed method not only in the task of reducing false alarm rate but also in improving the accuracy of APT IP prediction results. Accordingly, the effectiveness of the FIERL model in the paper is due to a flexible combination of the IP feature synthesis and extraction method based on the flow network and advanced computational techniques in representation learning. Specifically, with the proposal to use the BiLSTM-Attention combined model, we have succeeded in representing the information of IP based on the flow network. Obviously, with the support of the BiLSTM network, the basic features of flow networks have been fully learned and extracted. Then, based on the Attention network, the IP features in the flow networks have been highlighted instead of just averaging. Thus, compared with other approaches, it is clear that the approach using the BiLSTM-Attention combined network has satisfied both conditions of efficiency and simplicity. And finally, based on an intelligent calculation combination of Dropout and Contrastive Learning, the FIERL model has shown its effectiveness compared to other approaches as well as other computational techniques such as Smote and Triplet loss. Based on the experimental results in scenario 2, we have proven the scientific correctness as well as the need to combine Dropout and Contrastive Learning algorithms. Thus, it can be seen that, with the number of flows in each IP being different, the problem is how a classification system can train and detect which IP belongs to an APT attack and which IP is normal. This is a really hard task. The APT attack detection approach based on the FIERL model is a novel, original, meaningful approach and no research has proposed it. From here, the use of deep learning networks combining Attention and Representation Learning can be considered as a new approach that gradually replaces the traditional approaches in the APT attack detection task. In addition, this approach has not only contributed to solving some difficulties of the APT attack detection task but also opened up new research directions and approaches for the task of detecting other anomalies such as malicious code, unauthorized intrusion, insider, etc. In the future, to improve the ability to detect APT IP, we think that we can consider improving and supplementing 3 main issues: i) the method for building and extracting abnormal behaviors of IP; ii) the rebalancing data method in imbalanced datasets; iii) the classification method by using unsupervised learning.

## Supporting information

S1 File (ZIP)
